# Laparoscopic resection of gastric duplication cysts in newborns: a report of five cases

**DOI:** 10.1186/s12893-017-0234-x

**Published:** 2017-04-12

**Authors:** Hong-Xia Ren, Li-Qiong Duan, Xiao-Xia Wu, Bao-Hong Zhao, Yuan-Yuan Jin

**Affiliations:** Department of Pediatric Surgery, Shanxi Children’s Hospital, 13 New People Avenue, Taiyuan, 030013 Shanxi China

**Keywords:** Gastric duplication cyst, Laparoscopic resection, Newborns

## Abstract

**Background:**

Gastric duplication cysts are rare congenital alimentary tract anomalies and most cases are recognized during childhood. There were few reports about gastric duplication cysts in newborns and even fewer reports about laparoscopic resection of gastric duplication cysts in newborns.

**Case presentation:**

We report a series of five newborns with gastric duplication cysts which were successfully resected by laparoscopy between January 2010 and April 2015. Case 1, a male newborn was admitted because of severe salivation, choking cough and dyspnea for 30 min after birth. Case 2, a male, was suspected of duodenal ileus by antenatal examination. Case 3, a female was admitted because of vomiting for 5 days. Case 4,a female without significant symptoms simply visited us for the abdominal cyst detected by antenatal examination. Case 5, a male was admitted because of vomiting for 4 days. All patients were performed with a surgery after assistant examinations. Case 1 was died of respiratory failure and the other patients recovered uneventfully.

**Conclusion:**

Gastric duplication cysts in newborns are very rare. Laparoscopic surgery play an important role on the diagnosis and treatment. Our experience and practice indicate that laparoscopic resection of gastric duplication cysts in newborns is viable and there is also a need to increase sample size to prove its safety and effectiveness.

## Background

Gastric duplication cysts (GDCs) are rare congenital alimentary tract anomalies and most cases are recognized during childhood. Nowadays, an increased number of cases of GDCs have been reported in newborns because of the accessibility of antenatal examination. Advancements in laparoscopic techniques and skills have made it possible to treat gastric duplication cysts in an earlier phase and with a minimal incision. We report five cases with gastric duplication cysts treated by laparoscopic surgery in newborns.

## Case presentation

**Fig. 1:**
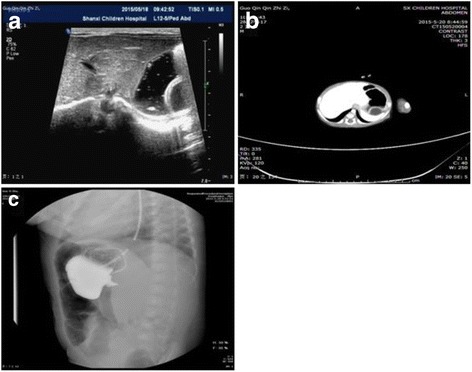
Ultrasound image (**a**), CT image (**b**);Pyloric obstruction by radiography (**c**)

Included in this report, two male and three female newborns aged between 1 h and 28 days with a mean of 14 days, weighing between 2.3 and 4.4 kg with a mean of 3.45 kg. Details of the five cases are shown in Table 1. Case 1, a male newborn was admitted as an emergency because of severe salivation, choking cough and dyspnea for 30 min after birth, and preoperative examination suspected him of esophageal atresia (Gross A) complicated with an abdominal mass. Case 2 was a male newborn who was suspected as having duodenal ileus by antenatal examination, and admitted because of double bubble sign detected by postpartum X-ray radiography. Case 3, a 26-day-old female was admitted to the hospital because of vomiting for 5 days. Case 4,a 28-day female without significant symptoms simply visited us for the abdominal cyst detected by antenatal examination. Case 5, a 14-day-old male newborn was admitted because of vomiting for 4 days. All patients underwent transabdominal color Doppler ultrasonography and upper digestive tract radiography. In Case 3 and 4, the patients also underwent computed tomography (CT) and magnetic resonance imaging (MRI) before a surgery. The Case 3 was originally suspected as common bile duct cyst, which was excluded by intraoperative cholangiography. Therefore, the diagnosis of GDCs was only confirmed pre-operative in Case 4 and 5. The ultrasound, CT, and upper gastroenterograpic images of the five patients are shown in Fig. 1.

**Table 1:** Clinical data of the four neonates with gastric duplication cyst

NO.	1	2	3	4	5
Gender	male	female	female	female	male
Age	1 h	1 day	26 d	28 d	14 d
Weight (kg)	2.8	2.7	4.2	4.3	3.9
Presentation	saliva bucking	emesis	Emesis	asymptomatic	emesis
Preoperative diagnosis	esophageal atresia, abdominal mass	gastric duplication?	choledochal cyst?	abdominal mass	gastric duplication?
Surgical procedures	Laparoscopic resection and gastrostomy	laparoscopic resection and repair stomach	Cholangiography and laparoscopic resection	laparoscopic resection	laparoscopic resection
size of mass (cm^3^)	2 × 3 × 3.5	2 × 3 × 2	5 × 4 × 3	2 × 3 × 4	2 × 2.5 × 2
Location of the mass	middle of greater curvature	near cardia	pylorus	antrum of pylorus	near cardia
Accompanied malformation	EA(Gross A), PDA, ASD	PDA, PDA	PFO	PFO	PFO
Prognosis	abandoned	cured	cured	cured	cured

*EA* esophageal atresia, *PDA* ventricular septa defect patent ductus arteriosus, *ASD* atrial septal defect, *PFO* patent foramen ovale

**Fig. 2:**
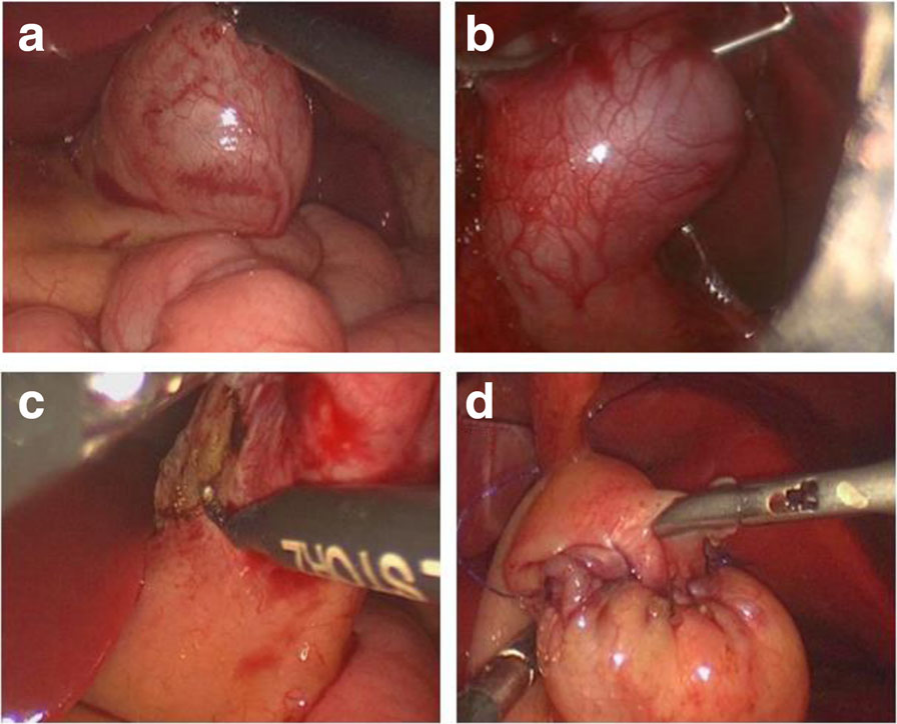
Mass located in the cardia (**a**); Percutaneous puncture (**b**); Mass resection (**c**); Suturing of the wall of stomach (**d**)

Laparoscopic resection of the gastric duplication cysts was performed with a 5-mm, 30° or 0° camera and three ports, including a 5-mm port near the umbilicus and two 3-mm ports. Using a 4-0 needle, the ligamentum teres hepatis was pushed upward to expose the surgical filed. Among the five cases, Case 3 was preoperatively misdiagnosed by cholangiography and intraoperatively diagnosed as gastric duplication cyst, with was excised subsequently. As described previously, the patient in Case 3 was originally suspected as having common bile duct cyst, but intraoperative cholangiography did not find significant abnormality in the common bile duct. Further laparoscopic exploration revealed the existence of GDC, which was resected thereafter. As it was very difficult to expose the cyst in the cardia with a surgical forceps in the left upper abdomen in case 2 (Fig. 2a), laparoscopy was performed to confirm the diagnosis, location, adhesion and relationship of the cyst with the adjacent organs. Fluid in the duplication cyst was aspirated by percutaneous puncture using a syringe to decrease the tension and volume of the mass before excising the cyst (Fig. 2b). During the operation, complete dissociation and excision of the cyst was performed along the serous membrane in Cases 1, 2 and 5 (Fig. 2c). In Case 2 the gastral cavity was exposed, and the wall of stomach was repaired with a 5-0 Coated Vicryl *Plus Antibacterial (ETHICON) (Fig. 2d). In Case 1, the wall of stomach was repaired by pouch suture and then raised to the abdominal wall for a gastric fistula because of the associated type I esophageal atresia. In cases 3 and 4, the cyst was mostly excised and the mucosa of the residual wall was cauterized. All the surgical procedures were completed successfully by laparoscopy. The patient in Case 1 was discharged three days after surgery because of the economic reason. Recoveries of the other four cases were uneventful without significant postoperative complications such as ileus, bleeding, anastomotic leakage, infection of incision or relapse.

## Discussion

Gastric duplication is a relatively rare congenital malformation, accounting for about 9% of all gastrointestinal duplications. Numerous theories have been proposed about the etiology of foregut duplication, including the segmental twinning theory, recanalization barrier of the alimentary tract, persistence of fetal gut diverticula, and separation barrier of the notochord and archenteron. Gastric duplication cysts can be found at any part of the stomach. The typical ones are usually located along the greater gastric curvature, and some others may be along the anterior or posterior wall of stomach, or located in the cardia or pylorus. Most cysts are single, elliptical, spherical, cystic, and linked with gastric muscular layers. Rowling et al. [1] suggested the pathological diagnostic criteria of gastric duplications as follows: lining with the gastrointestinal mucosa, attachment to the gastrointestinal tract, the presence of a smooth muscle coat, communicating with gastric lumen or not, and sharing blood with the stomach.

Symptoms of gastric duplications are atypical and often present with gastrectasia, an abdominal mass and vomiting. Larger cysts can cause abdominal pain and discomfort; larger tension may cause stomach mucosa bleeding and vomiting; and those located in the cardia or pylorus may present with obstruction. Patients with gastric duplications were usually diagnosed because of abdominal pain, vomiting and abdominal swelling before 2 years old [2], while some cases were found in adulthood [3, 4]. Diagnostic work-up includes x-ray, ultrasound, CT and MRI. Hlouschek [5] reported a case of gastric duplication in an adult by endoscopic ultrasonography. Gastric duplication is so rare that it is often misdiagnosed as pancreatic cyst, adrenal cyst, or stomach muscle adenoma for that the muscle layer will be shared by the cyst and stomach . There was a reported case which the cyst located in the posterior gastric wall was preoperatively misdiagnosed as renal cyst [6]. In our series, Case 3 was misdiagnosed as choledochal cyst by MRI and later intraoperatively confirmed as a gastric duplication cyst, which reminded us of the possibility of a gastric duplication cyst located in the pylorus. To establish a definitive diagnosis, abdominal X-ray radiography and/or CT is often necessary for newborns or infants who present with vomiting, hematemesis in particular, bloody stools, and a palpable abdominal mass, although the definite diagnosis of gastric duplication cysts finally depends on surgery and pathology. In addition, The cyst lining may undergo ulceration, erosions, regenerative and increased fluid production pressure-induced in noncommunicating cysts may result in necrosis of the mucosa, which may cause bleeding into the cyst or perforation into the peritoneal cavity. Besides duplication cysts have the potential for neoplastic transformation.

Machado et al. [7] first reported laparoscopic resection of gastric duplication, followed by Tayar et al. [8–11]. However, laparoscopic resection of gastric duplication cysts in newbrons is rarely reported. At present, laparoscopy has been used in newborns with choledochal cyst, duodenal obstruction and esophageal atresia. For gastric duplication cysts, laparoscopy can not only confirm the diagnosis but also avoid abdominal laparotomy, thus decreasing possible traumatic injury. All the five newborn patients in our series underwent gastric duplication cyst resection by laparoscopy, including one who even underwent a gastrostomy. Technically, all the five cases achieved satisfactory outcomes. So we believe that laparoscopy is quite safe, effective, and feasible in the diagnosis and treatment of gastric duplication cysts, and should also be applicable to detect other gastrointestinal multiple malformations. Compared with conventional surgery, laparoscopy is characterized by less trauma, more acceptability on the part of both the patient and the doctor, and quicker postoperative recovery. The Case 1 gave up treatment and died of respiratory failure. The other four patients recovered uneventfully without significant postoperative complications and grew up well with no significant difference as compared with average children of the same age during the follow-up period.

## Conclusions

Gastric duplication cysts in newborns are very rare. Laparoscopic surgery play an important role on the diagnosis and treatment. Our experience and practice indicate that laparoscopic resection of gastric duplication cysts in newborns is viable and there is also a need to increase sample size to prove its safety and effectiveness.

## Abbreviations

CT: Computed tomography
GDCs: Gastric duplication cysts
MRI: Magnetic resonance imaging
